# Association of specific HLA alleles in patients with interstitial cystitis suggesting autoimmunity

**DOI:** 10.3389/fmmed.2025.1712660

**Published:** 2025-12-04

**Authors:** Inna Tabansky Stern, Jiayao Wang, Robert M. Moldwin, Jason M. Kim, Jason H. Singh, Derek C. Tran, Souhel Najjar, Melis Akinci, Alexis Howard, Joseph E. Duke-Cohan, Marwa Belhaj, Jonathan Stevens, William J. Lane, Lori A. Birder, Edwin K. Jackson, Derin B. Keskin, Guanglan Zhang, Joel N. H. Stern

**Affiliations:** 1 Department of Urology, Renaissance School of Medicine at Stony Brook University, Stony Brook, NY, United States; 2 Northwell Health, The Smith Institute for Urology, New Hyde Park, NY, United States; 3 Institute of Molecular Medicine, The Feinstein Institute for Medical Research, Manhasset, NY, United States; 4 Department of Biomedical Informatics and Department of Systems Biology, Columbia University, New York, NY, United States; 5 Department of Urology, Donald and Barbara Zucker School of Medicine at Hofstra/Northwell, Hempstead, NY, United States; 6 Department of Neurology, Donald and Barbara Zucker School of Medicine at Hofstra/Northwell, Hempstead, NY, United States; 7 Department of Neurology, Lenox Hill Hospital, New York, NY, United States; 8 Translational Immunogenomics Laboratory, Department of Medical Oncology, Dana-Farber Cancer Institute, Boston, MA, United States; 9 Department of Pathology, Brigham and Women’s Hospital, Boston, MA, United States; 10 Department of Medicine, School of Medicine, University of Pittsburgh, Pittsburgh, PA, United States; 11 Health Informatics Lab, Department of Computer Science, Metropolitan College, Boston University, Boston, MA, United States; 12 Section for Bioinformatics, Department of Health Technology, Technical University of Denmark, Lyngby, Denmark

**Keywords:** IC/BPS, HIC, NHCI, HLA, Autoimmunity

## Abstract

Interstitial cystitis/bladder pain syndrome (IC/BPS) with Hunner Lesions (Hunner Type Interstitial Cystitis or HIC) is characterized by lesions on the bladder wall. Previous work on these lesions identified B cells and monocytes within the lesion. However, the overall role of the adaptive immune system in the disorder remains uncertain. In this study, we performed HLA sequencing on 12 IC/BPS patients with HIC and 7 Non Hunner Type IC (NHIC) patients, and identified HLA-DQB1*02:02 and HLA-DRB1*07:01:01 have a significant association with HIC. This pilot study provides genetic evidence supporting a potential autoimmune component in HIC and may help define the pathogenesis of at least one subtype of IC/BPS, and lay the groundwork for identifying the etiology of IC/BPS as a disease complex. Identifying the mechanisms can also open new approaches to treatment. Identifying an HLA haplotype associated with HIC would indicate that it is autoimmune.

## Introduction

Interstitial cystitis/bladder pain syndrome (IC/BPS) is defined as pelvic pain with bladder filling and irritative voiding symptoms for at least 6 weeks without the presence of infection or other causative urologic conditions (Nickel and Moldwin, 2018). Most patients’ bladders show only mild inflammation on cystoscopy, while a subset of patients present with highly visible regions of inflammation and ulceration on the bladder wall. These regions of inflammation are known as Hunner lesions (Whitmore et al., 2019). These patients frequently have a more severe clinical course, but the two forms of IC/BPS rarely, if ever, interconvert. This form of IC/BPS is known as IC/BPS with Hunner lesions or Hunner Type IC (HIC). The more common form of IC/BPS is without Hunner lesions, also known as Non Hunner Type IC (NHIC).

There are several treatment modalities for IC/BPS, including surgical fulguration of Hunner lesions, corticosteroids and hydro distention of the bladder however, treatment efficacy is frequently limited (Hanno et al., 2015). Diagnosis of IC is also frequently a challenge due to the variable presentation and lack of biomarker availability. Patients are often diagnosed first with recurrent UTI, although the cause and effect relationship between UTI and IC/BPS is unclear. It is possible that recurrent UTIs may contribute to the development of IC/BPS. Alternatively, IC/BPS may predispose patients to UTIs or mimic UTIs by producing UTI-like symptoms due to bladder inflammation and disruption of mucosal barriers.

While the etiology of IC/BPS remains uncertain, there is growing evidence that activation of the immune system is involved in the disease. For instance, B cells have long been observed in Hunner lesions (HL), and a recent histological study showing that they are enriched within the lesion (Moldwin et al., 2022). When B-cell receptors in HL were sequenced, strong evidence emerged that they respond to the same antigen in multiple patients and proliferate within the lesions, as different B cell clones were observed to have the same B cell receptor (Tabansky et al., 2022a). These observations indicate that they are likely responding to a specific antigen in that location. Recent research has identified B cells expanding in response to a specific, unidentified antigen within Hunner lesions of IC patients (Tabansky et al., 2022a). These findings strongly suggest a targeted adaptive immune response within these lesions, consistent with either an autoimmune etiology or a causative antigen.

While multiple studies indicate that immune cells are involved in the processes underlying bladder inflammation in IC, none of them provide direct evidence of autoimmunity. The experiments conducted thus far cannot distinguish whether immune cells play a protective role in IC (for instance, if it is caused by an unidentified pathogen or chemical damage to the bladder wall) or whether they contribute detrimentally by causing inflammation.

There is evidence that susceptibility to IC/BPS is heritable, as suggested by twin studies and SNP association studies (Warren et al., 2001; Altman et al., 2011; Cassão et al., 2019). Heritability would indicate that the disease has a genetic predisposition in addition to environmental factors. If similar alleles are found in multiple patients with IC/BPS as compared to the population overall, the function of the proteins encoded by these alleles would yield clues to the mechanism of the disease. Knowing the mechanisms of disease helps design more specific and targeted treatment and identify biomarkers.

Association with Human Leukocyte Antigens (HLA) has long been considered the gold standard of evidence that a disease is autoimmune. Some preliminary evidence has emerged in recent literature that there may be an HLA association with IC/BPS. Still, these findings were incidental to the studies, and therefore, many studies were not optimally designed to probe that question. For instance, a recent GWAS study in a Japanese population of IC/BPS patients found an association between IC/BPS and the SNPs in the genomic locus containing the human major histocompatibility complex (MHC), particularly HLA-DQB1 (Akiyama et al., 2023). This study used regression analysis to identify the possible HLA association and, therefore, could not identify specific HLA alleles involved in IC/BPS, as greater sequencing depth and accuracy are required for HLA typing due to the complex nature of this genetic locus.

To explore whether HIC has an autoimmune component, we asked whether certain HLA alleles are more common in patients with HIC than in those without lesions or in healthy populations. To test this hypothesis, we performed high-resolution HLA sequencing in patients carefully stratified into HIC and NHIC groups based on clinical presentation. Comparing allele frequencies across these groups and with the general population allowed us to identify alleles enriched in HIC that may point to an immune-mediated form of the disease and provide a genetic basis for future diagnostic and therapeutic advances.

## Materials and methods

### Patients and sample collection

All research was conducted with the approval of our institutional review board. Patients were recruited from the urology clinic. All patients included in the study presented with IC/BPS as defined by NIDDK guidelines and were determined to have HL on cystoscopic examination (Gillenwater and Wein, 1988). Given the rarity of HIC, the present cohort includes all eligible patients available during the collection period, representing approximately 25% of all IC/BPS cases seen in our clinic over 2 years. The sample size was determined by patient availability rather than a formal power calculation, consistent with exploratory genetic association studies of rare disorders. Blood samples were collected from selected patients in accordance with protocols approved by our IRB. Written informed consent was obtained from all participants.

### High resolution HLA typing

NGS HLA typing is performed using the AlloSeq Tx 17 system (CareDx, Inc. Brisbane, CA). Whole genome DNA libraries are prepared using bead-bound transposons followed by sample indexing and pooling. HLA genes are captured using biotinylated probes providing full gene coverage for HLA class I genes and full exon coverage for HLA class II genes. Libraries are enriched and prepared for sequencing on the Illumina MiSeq (Illumina, San Diego, CA). The sequence data is analyzed using AlloSeq Assign software (CareDx, Inc. Brisbane, CA) and HLA genotypes are assigned using 3 field HLA nomenclature (Jiang et al., 2025).

### Genotype frequency and haplotype frequency tests

Sequenced frequency was compared with control data from the National Marrow Donor Program (NMDP) registry. If the identified allele was not present in the database, we searched them in the literature (Varadi et al., 2022; 2024). For example, HLA-DQB1*02:02 genotype and allele frequency was used from Murray et al. (2007). Association between each allele and IC/BPS was conducted using the Fisher exact test. Allele frequency was tested for each allele sequenced. Genotype frequency was tested if the genotype is available for control data.

## Results

### HLA association with IC/BPS

**FIGURE 1:**
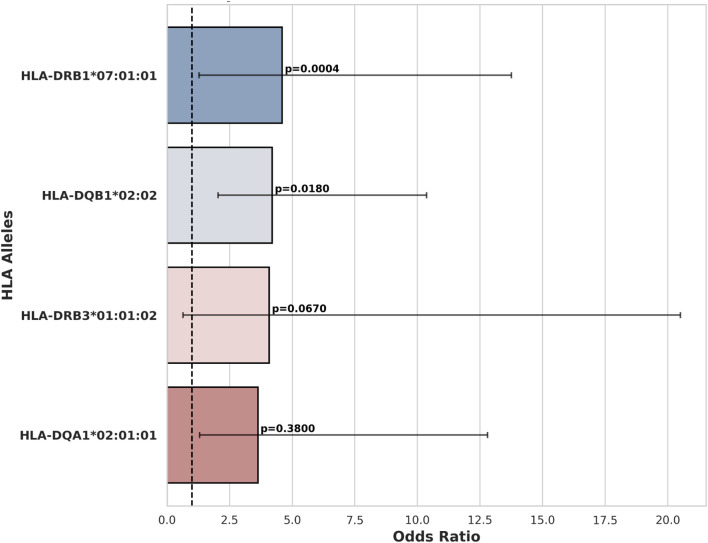
HLA alleles associated with HIC.

**FIGURE 2:**
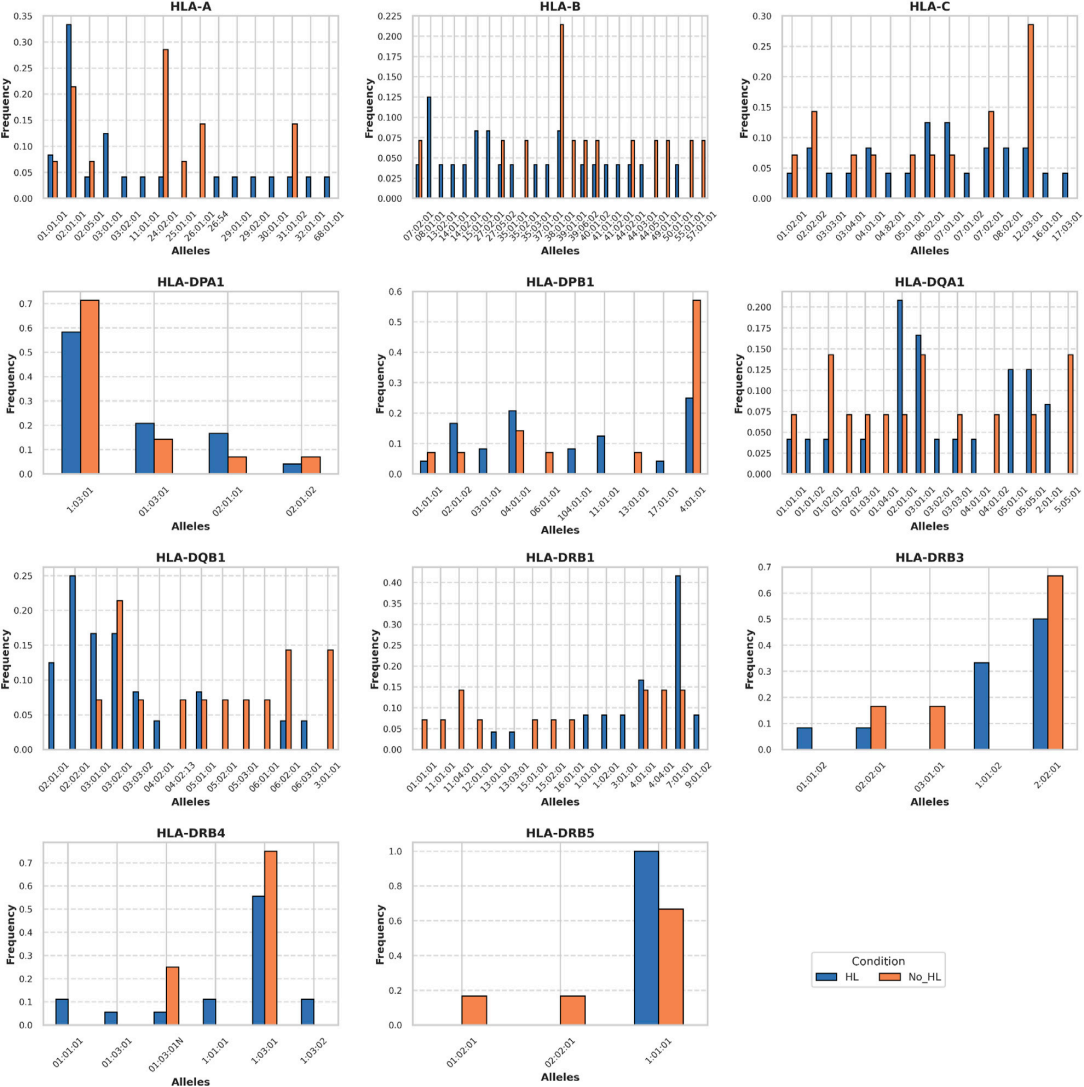
Allele frequency of different HLA alleles found in our cohort of HIC and NHIC patients.

We sequenced HLAs of 12 patients with HIC and 7 patients with NHIC. In 6 of the 12 (50%) patients that underwent high resolution HLA typing, we identified a rare allele, HLA-DQB1*02:02. HLA-DQB1*02:02 allele has no recorded frequency in the European population and its descendants, as the allele is absent from the NMDP database (Berman et al., 2000). We identified a documented occurrence of this allele in a study on celiac disease (Murray et al., 2007). Compared to reported health control genotype frequency in Murray et al. (14 out of 102 healthy controls), HLA-DQB1*02:02 allele has a significant association with HIC (odds ratio = 6.29, P-value = 0.0065) (Figures 1, 2) HIC patients (Table 1) also have higher frequency compared to NHIC patients (Table 2), where 0 out of 7 had DQB1*02:02 (odds ratio = inf, p-value = 0.4).

**TABLE 1:** Allele frequency and phenotype frequency of the HLA-DRB1 allele for patients with HIC. Highest allele frequency and phenotype frequency values are highlighted.

HIC patients	HLA-DRB1	Allele frequency	Phenotype frequency
IC-39	1:01	0.14	0.27
IC-22	1:02	0.14	0.27
IC-43	1:02	0.14	0.27
IC-24	3:01	0.14	0.27
IC-82	3:01	0.14	0.27
IC-120	3:01	0.05	0.27
IC-24	4:01	0.18	0.36
IC-31	4:01	0.18	0.36
IC-44	4:02	0.18	0.36
IC-45	4:03	0.18	0.36
IC-9	7:01	0.23	0.36
IC-9	7:01	0.23	0.36
IC-39	7:01	0.23	0.36
IC-44	7:01	0.23	0.36
IC-45	7:01	0.23	0.36
IC-31	8:05	0.05	0.09
IC-82	9:01	0.05	0.09
IC-43	11:01	0.05	0.09
IC-22	12:01	0.05	0.09
IC-42	13:01	0.09	0.09
IC-42	13:03	0.09	0.09
IC-120	15:01	0.05	0.09

**TABLE 2:** Allele frequency and phenotype frequency of the HLA-DRB1 allele for patients with NHIC. Highest allele frequency and phenotype frequency values are highlighted.

NHIC patients	HLA-DRB1	Allele frequency	Phenotype frequency
IC-11	1:01	0.07	0.14
IC-5	4:01	0.21	0.43
IC-10	4:02	0.21	0.43
IC-1	4:04	0.21	0.43
IC-6	7:01	0.07	0.14
IC-6	8:04	0.07	0.14
IC-108	11:01	0.14	0.29
IC-10	11:04	0.14	0.29
IC-108	12:01	0.07	0.14
IC-1	14:54	0.07	0.14
IC-5	15:01	0.21	0.43
IC-11	15:01	0.21	0.43
IC-2	15:02	0.21	0.43
IC-2	16:01	0.07	0.14

HLA-DQB1*03 alleles are also prevalent in patients with HL, however, at the current sample size, we did not identify a strong association (odds ratio = 3.5, P-value = 0.13). We also observed that the HLA-DRB1*07:01:01 allele has a significantly higher allele frequency (AF = 0.41) in our HIC cohorts than the allele frequency (AF = 0.13) reported in DMBD (odds ratio = 4.6, P-value = 0.0004). Other alleles have higher allele frequency than general populations, including HLA-DRB3*01:01:02 and HLA-DQA1*02:01:01. However, the association is not significant, possibly due to the small sample size of our patient cohort. Thus, the specific HLA allele we have observed is strikingly over-represented in our randomly selected patients. Additional sequencing studies and analysis of more samples will provide sufficient statistical power to determine whether this result is significantly different from the general population.

## Discussion

There is considerable evidence that IC/BPS is a systemic disorder. These multiple lines of evidence include the association with other diseases, the inflammatory nature of the changes encountered in the bladder, and frequent comorbidity with other pain (Hanno et al., 2015). Multiple studies now indicate that the adaptive immune system is involved in HIC. B cells responding to antigens have been observed in Hunner lesions, but it is unclear whether these cells serve a protective role or a precipitating role in HIC. HLA sequencing and HLA association are the gold standard of evidence for determining whether a disorder is autoimmune. The present study addresses this question by conducting deep sequencing of HLA regions in patients with IC/BPS in a case-control design.

The present study has several limitations, including a small sample size. This reflects the rarity of HIC and the limited number of eligible patients available during the study period; the current cohort includes all patients who met the inclusion criteria at our institution. Although the sample size constrains statistical power, the observed enrichment of specific HLA alleles suggests a genuine biological signal that warrants further validation. Future multi-center studies with larger and more diverse cohorts of HLA sequencing will be essential to confirm these associations and assess their generalizability across populations. Despite the small sample sizes of NHIC and HIC populations, we were able to identify an HLA allele significantly enriched in HIC patients. The patients analyzed in the present study were Americans of European descent; it is important to reproduce these results with people of different origins to determine whether the HLA association with HIC is local or global. It is possible for autoimmune diseases to be associated with different HLA alleles in different populations. For instance, neuromyelitis optica spectrum disorders have shown associations with different HLA alleles depending on the geographic region from which patients originate (Tabansky et al., 2022b). HLA-DQB and HLA-DQA encode a heterodimer that is expressed on antigen-presenting cells and used to present foreign antigens to immune cells. The B1 allele specifically forms the binding pocket which complexes to the antigen. The overall frequency of HLA DQB1*02 allele in multiple populations where it has been measured is approximately 5% (Krini et al., 2012). The HLA-DQB1*02:02 allele was previously associated with celiac disease, SLE, and type 1 diabetes mellitus (Mosaad et al., 2012; Cabrera et al., 2018). In patients with celiac disease, this allele has been reported at a frequency of approximately 20% of the population (Krini et al., 2012). HLA-antigen binding. HLA-DQB1*02:02 is a rare allele associated with celiac disease (CD), along with HLA-DQA1*02:01. These two form an MHC II heterodimer, HLA-DQ2.2, that presents gluten epitopes (Ting et al., 2020). DQ2.2-glut-L1 is the predominant antigen that binds to HLA-DQ2.2 with a sequence of PFSEQEQPV. P4-Glu, P6-Glu, and P7-Gln form an extensive network of hydrogen bonding with Arg70β, Lys71β, Tyr9β, and Ser30β. Negatively charged or polar residues at P4, P6, and P7 contribute to stronger MHC-antigen binding. Bulkier hydrophobic groups at P1 and P9 likely stabilize the interaction (Ting et al., 2020).

A notable finding from the analysis described here is the difference in HLA association between HIC and NHIC forms. While currently classified as the same disease, there is accumulating evidence on multiple fronts that HL are a separate disease process unique to HIC. For instance, a prior study from our lab has already established a significant clonal proliferation of plasma cells within Hunner’s lesions, which is not present in NHIC bladder mucosa (Tabansky et al., 2022a). Recent immune profiling studies corroborate these findings, with elevated transcription of genes encoding BAFF and April, which are key regulators of clonal B-cell expansion (Horie et al., 2024). There is also evidence of macrophage dysregulation in HIC compared to NHIC, with an elevated fraction of M1 macrophages and a reduced fraction of M2 macrophages, as well as their associated anti-inflammatory and immunosuppressive factors (Ko et al., 2024).

Multiple pre-clinical animal models are available to evaluate aspects of IC/BPS physiology. These animal models mimic various aspects of the disease, including: disrupting bladder mucosal barriers with caustic chemicals, inducing chronic inflammation of the bladder with intravesical treatment, and experimental autoimmune cystitis (Tay and Grundy, 2023). All these models have potential to mimic different clinical aspects of IC/BPS and to explore treatment options. Most relevant to addressing the autoimmune etiology of IC/BPS is the autoimmune cystitis approach, which induces true autoimmunity against the bladder in rodents. There are three models available to allow autoimmune cystitis to develop. One model included injection of bladder homogenate from mice subcutaneously into the bladder of unrelated mice to induce autoimmunity against bladder antigens in a non-specific manner (Jin et al., 2017; Liu et al., 2019). Alternatively, a model that uses uroplakins (Lee, 2011), bladder urothelial proteins has also been observed to induce adaptive immune responses with increased urinary frequency in mice (Altuntas et al., 2012). Finally, a model using transgenic mice is also available, where the urothelium expresses a recombinant protein ovalbumin, and immunity is induced by transfer of T cells from another mouse with immune response to that protein (Liu et al., 2007). Finally, a natural model exists, as a disorder similar to interstitial cystitis has been documented in domestic cats (Birder et al., 2003). FIC is most similar to NHIC based on histologic evidence (Jones et al., 2021). It has the advantage of being a natural idiopathic condition mimicking this aspect of IC/BPS but cannot be induced in the lab. As animal models are central to preclinical drug development, an important factor that remains to be determined is whether the available models of autoimmunity will sufficiently mimic the mechanisms of human IC/BPS pathogenesis to allow preclinical drug development. The availability of multiple, thoughtfully designed animal models of autoimmunity makes it more likely that at least one of them will be a good reflection of human pathology and become a useful tool for preclinical development of novel therapies.

An autoimmune etiology in HIC that is distinct from NHIC would strongly determine how these diseases are managed. Currently, HIC and NHIC can be managed through intravesical therapy, such as hydrodistension and instillation of DMSO (Hanno et al., 2015). However, targeted anti-interleukin therapies like Stelara (ustekinumab) or TNF-alpha blockers like Humira (adalimumab) are used to manage autoimmunity. It is an intriguing thought that intravesical instillation (or even intravesical injection) of these treatment agents could provide relief to patients with IC/BPS. Prior randomized controlled trials have been conducted with TNF-alpha blockers, including certolizumab pegol and adalimumab, to treat patients with severe refractory IC/BPS (Bosch, 2018; Bosch, 2014). These trials demonstrated a statistically significant reduction in bladder pain and urinary frequency, but patients were not stratified into HIC vs. NHIC, and a significant placebo effect was reported (Bosch, 2014). Thus, future trials with phenotypic categorization and longer follow-up should be conducted. Monoclonal antibodies against BAFF, like belimumab, should be considered, given the evidence of clonal B-cell expansion in Hunner lesions and the drug’s known efficacy in managing systemic lupus erythematosus. Now that the HLA has been identified more work can be done to locate the potential antigen that drive the cellular responses associated with IC/BPS.

## Data Availability

The datasets presented in this study can be found in online repositories. The names of the repository/repositories and accession number(s) can be found in the article/Supplementary Material.
