# Supplementary material for: Association of specific HLA alleles in patients with interstitial cystitis suggesting autoimmunity
Source: Front Mol Med. 2025 Dec 4;5:1712660. doi: 10.3389/fmmed.2025.1712660 (PMC12712699; doi:10.3389/fmmed.2025.1712660)
Supplement: Supplementary file 1 [file Table1.docx]

Supplementary Figures

|  | N | Sex distribution (No. Males/No. Females) | Avg. Age | Average IPSS Score | Average GUPI Score |
| --- | --- | --- | --- | --- | --- |
| HIC | 11 | 2/9 | 63.0 | 17.4 | 25.0 |
| NHIC | 7 | 1/6 | 48.1 | 14.2 | 21.6 |

**Supplementary Figure 1**. Clinical characteristics and sex breakdown of 2 research cohorts displaying either Hunner Type IC (HIC) or non-Hunner Type IC (NHIC). Patients signed consent forms and filled questionnaires. Questionnaires included an IPSS form and a GUPI form where scores were averaged for each group.
